# Human Papilloma Virus Awareness Among Hispanic Females with Inflammatory Bowel Disease

**DOI:** 10.1007/s40615-015-0112-0

**Published:** 2015-04-22

**Authors:** José E. Rivera-Acosta, Maysabel Aponte, Irene Villamil, Josefina Romaguera, Ana P. Ortiz, Esther A. Torres

**Affiliations:** Division of Gastroenterology, Department of Medicine, University of Puerto Rico School of Medicine, PO Box 365067, San Juan, PR 00936-5067 Puerto Rico; University of Puerto Rico Comprehensive Cancer Center, San Juan, Puerto Rico

**Keywords:** Human papilloma virus, Inflammatory bowel disease, HPV vaccine, Cervical cancer

## Abstract

Women with inflammatory bowel disease (IBD) may be at increased risk of human papilloma virus (HPV) infection and HPV-related malignancies, as many are immunocompromised secondary to the use of immunosuppressant agents. Several studies have addressed the knowledge about cervical cancer risk factors in different populations, particularly HPV infection and its association with cervical malignancies; most of these studies show poor patient knowledge. The purpose of this study is to describe the knowledge of females with IBD about HPV infection and the HPV vaccine. We performed a cross-sectional study in 147 consecutive patients attending the clinics of the University of Puerto Rico Center for IBD from 2009 to 2010. An interviewer-administered questionnaire was used to collect information on demographics, lifestyles, and HPV-related knowledge of participants. Bivariate analysis using the chi-square statistics and Fisher’s exact test was used to examine factors associated with HPV awareness. The mean age of participants was 36.6 years (SD = 13.91 years). Three fourth (77 %) of women had awareness of the existence of HPV, and 58 % did know about the existence of HPV vaccines. Among those who had heard about HPV, 79.6 % knew that HPV can cause cervical cancer, and 57.5 % knew that the virus is sexually transmitted. Among those who knew of the vaccine, 75.3 % learned about its existence through the media, while only 15.3 %, through their health-care provider. Only three women within recommended ages (2 %) had been vaccinated against HPV, although 50 % of participants indicated that they would definitely/probably vaccinate against HPV in the future. A significant trend was observed, where more educated women were more likely to have heard of HPV (*p* for trend = 0.0017). Women who were high school graduates/some college (OR = 6.63, 95 % CI = 1.71–25.66) and those with at least an associate degree (OR = 11.69, 95 % CI = 3.05–45.89) were more likely to be aware of the HPV vaccine than women without a high school degree. Our study documents poor knowledge of HPV and HPV vaccine in this population of IBD patients in Puerto Rico. Although vaccination coverage is low in this population, women are receptive to the possibility of vaccination in the future. Given that this population may be at an increased risk of HPV infection and related morbidities, education and vaccination programs should be promoted among them.

## Introduction

Human papilloma virus (HPV) is the primary etiologic agent for the development of cervical cancer and cervical dysplasia, along with other premalignant and malignant conditions of the vagina, vulva, and cervix. HPV has been also associated to anorectal, oropharyngeal, and head and neck cancer. It is estimated that 20 million Americans are infected with this virus, and a recent study has calculated the prevalence in women aged 14–59 years at 42.5 % [1]. Several studies have found that patients with inflammatory bowel disease (IBD) have a higher risk of developing cervical dysplasia [2, 3] compared with healthy women. IBD consists of several chronic inflammatory conditions that affect the gastrointestinal tract, being Crohn’s disease and ulcerative colitis the most frequent. These patients often require immunosuppressive therapy to control symptoms and disease progression. The risk of cervical dysplasia in IBD patients might be related to the use of immunosuppressive therapy in a high percent of these patients.

Cervical cancer has a major impact in females, particularly in developing countries where it is one of the leading causes of cancer death annually. According to recent estimates, 530,000 new cases of cervical cancer occur each year in women and 275,000 died of this disease in 2008 [4]. Latin America has been recognized as an area of high incidence rate of cervical cancer. The mortality rate associated with this malignancy is higher in Latin American countries when compared with other developed countries. This malignancy is potentially preventable by the establishment of screening programs that detect early cervical abnormalities and, more recently, by the availability of vaccination against HPV. Two HPV vaccines (Gardasil® and Cervarix®) were approved on 2006 as an effective way for primary prophylaxis of cervico-vaginal malignancies. These HPV vaccines target HPV 16 and 18 serotypes, which account for approximately 70 % of all cervical cancers [5]. In view of the proved efficacy of vaccination, the Pan American Health Organization endorsed the implementation of HPV vaccination on Latin America and the Caribbean in 2008 [6]. Few studies have assessed HPV vaccination rates in Latin America or the Caribbean, and there is no population-based study assessing HPV vaccination rate in Puerto Rico. A review article by Luciani et al. in 2011 found that compliance with the vaccination rate for the three recommended doses is only 27 % in the USA compared with 90 % for only the first shot [7]. Among the possible reasons for HPV vaccination rate incompliance are financial constraints, cultural belief about prophylaxis of sexually transmitted disease, poor access to health-care services, and poor knowledge and awareness about HPV and cervical cancer risk.

Several studies have demonstrated poor knowledge about HPV and its association with cervical cancer in the Hispanic population [8–10]. Hispanic women in the USA have the highest cervical cancer incidence rates compared with other ethnic groups [11]. Despite being a high-risk population, there is no study about the knowledge and attitudes about HPV infection and other cervical cancer risk factors in Hispanic women with IBD. In a high-risk population, determining patient knowledge and attitudes will help in the design of health-directed initiatives that positively impact prevention, prophylaxis, and surveillance.

In view of the close medical follow-up demanded for patient with chronic inflammatory conditions frequently requiring immunosuppressive medication, it would be expected that young women with IBD were more aware of routine health maintenance medical care, such as HPV vaccination and cervical cancer prevention. This study describes the knowledge about HPV infection and cervical cancer risk factors in a cohort of Hispanic women with IBD. Factors associated with HPV awareness and HPV vaccine awareness were analyzed.

## Methods

This cross-sectional descriptive study was performed at the UPR Center for Inflammatory Bowel Diseases clinics from February 2009 until June 2010. The study had approval from the University of Puerto Rico Medical Sciences Campus Institutional Review Board. The research coordinator identified potential participants among patients attending the clinics. All women older than 15 years with established diagnosis of IBD evaluated at the clinic were invited to participate in the study. From approximately 170 subjects invited to participate, 147 (86.5 %) women agreed to participate and were recruited. All study participants provided informed consent at the clinic. For subjects under 21 years old, informed consent was obtained from both the subject and the parents or legal guardian. After consent, research personnel administered a survey to the study participants at the clinic. The survey was administered in Spanish, the native language of all the participants. Estimated time of completion of the survey was 25 min. Surveys were identified with a code to protect confidentiality. The survey included questions about demographic information (age, place of birth, household income, education level, and civil status), lifestyle (use of tobacco), medical history (health maintenance, past medical diseases, history of sexually transmitted diseases), reproductive health (number of pregnancies, age at first pregnancy, age at first coitus, last menses, previous Pap smear results, history of abnormal Pap smear, use of contraceptive methods), sexual practices (age at first sexual encounter, number of partners), knowledge about HPV and other sexually transmitted diseases (STDs), and HPV vaccination history (knowledge about HPV vaccine, vaccine administration, etc.). In the education groups, participants were classified into those who did not complete high school education (no high school degree), those who completed high school but did not complete a college degree (high school graduate/no college degree), and those who obtained a college degree and/or completed postgraduate education (college degree or above). Civil status was classified as those who reported being single and in no romantic relationship, those married or in a romantic relationship, and those divorced or widowed. HPV awareness was evaluated with questions as follows: ‘Have you ever heard about the Human Papilloma Virus?’ (yes or no). HPV vaccine awareness was evaluated with the following question: ‘Have you ever heard about the existence of a Human Papilloma Virus vaccine?’ (yes or no). Other statements that reflected knowledge about symptoms of infection with HPV or cervical cancer and lifestyles that may increase the risk of HPV infection were also evaluated. This survey is a modification of a survey used by Colón-Lopez et al. to assess HPV awareness and knowledge on a Hispanic population [12].

Data from the study was recorded in a database designed with the use of Epi Info™, version 3.3.2. [13]. Statistical analyses were performed using SAS® version 8e for Windows [14]. Frequency distributions for categorical variables and summary measures for continuous variables were performed to describe the data. Chi-square and Fisher’s exact tests were used to determine the bivariate associations of demographic, age, socioeconomical status, education level, and participants’ HPV awareness and HPV vaccine awareness [15]. Multivariate logistic regression models were also used to assess these associations. Interaction terms within the logistic regression model were evaluated with the likelihood ratio test [16].

## Results

The mean age of participants was 36.6 years (SD = 13.91 year). Demographic characteristics are outlined in Table 1. Most of the participants reported to be born in Puerto Rico (91.8 vs. 8.2 % in USA); however, all participants were of Puerto Rican ethnicity. Overall, 53.0 % of the participants had Crohn’s disease, 42.9 % had ulcerative colitis, and 4.1 % indeterminate IBD. Overall, 86.5 % of the participants reported history of sexual intercourse, 19.9 % had an age of first coitus <16 years, and 8.2 % had a history of STDs. Approximately 20 % of the participants reported no previous Pap smear, and 18 % reported being diagnosed with an abnormal Pap smear in their lifetime. From all the 147 participants, almost one fourth (23.1 %) of the women (34 participants) had never heard of HPV, and 42.2 % did not know about the existence of HPV vaccines. Table 2 shows the percent of statements answered correctly by participants about HPV general knowledge and risk factors for HPV infection. Those participants who reported awareness about HPV (*n* = 113) answered general questions about HPV knowledge and risk factors. Among those who had ever heard about HPV, 79.6 % knew that HPV can cause cervical cancer and 57.5 % knew that the virus is sexually transmitted. Only 31.0 % knew that genital warts are caused by HPV while 19.5 % of the participants erroneously associated genital warts with herpes virus. A considerable percentage of the participants knew about risk factors for HPV infection. However, only half of the participants recognized that first coitus before the age of 16 years is an important risk factor for HPV infection. Most of the participants identified having multiple sexual partners and unprotected sex as important risk factors.

**Table 1:** Demographic characteristics of study participants (*N* = 147)

	Frequency	Percent
Age (years)		
≤35	80	54.4
≥36	67	45.6
IBD type		
Crohn’s	78	53.0
Ulcerative colitis	63	42.9
Indeterminate colitis	6	4.1
Education		
No high school degree	18	12.2
High school graduate/no college degree	57	38.8
College degree or above	72	49.0
Civil status		
Single	55	37.4
Married/in romantic relationship	73	49.7
Divorced/widow	19	12.9
Employment status		
Employed	55	37.41
Unemployed	72	48.98
Student	20	13.6
Socioeconomic status^a^		
<$24,000	83	56.8
$25,000–$99,000	20	13.7
>100,000	19	13.0
Unknown	24	16.4
Place of birth		
PR	135	91.8
USA	12	08.2
Parity^a^		
0	58	39.7
1–2	47	432.2
≥3	41	28.1
Lifetime sexual partners^c^		
0	19	13.5
1–2	81	57.4
≥3	41	29.1
Age of first coitus^b^		
<16	25	19.9
16–21	57	45.2
>21	44	34.9
Abnormal Pap		
Yes	27	18.4
No	91	61.9
No previous Pap	29	19.7
STD history^a^		
Yes	12	8.2
No	134	91.8
HPV vaccination history		
Yes	3	2.0
No	143	97.3
Don’t know	1	0.7
HPV vaccination participants 9–26 years old^d^		
Yes	3	5.4
No	53	94.6

^a^ *n* = 146; one participant refused to answer

^b^ *n* = 126; 21 participants refused to answer or had no history of sexual intercourse

^c^ *n* = 141; six participants refused to answer

^d^ *n* = 56; number of participants between 9 and 26 years old

**Table 2:** Percent of correct answered statement related to HPV knowledge in participants who reported awareness of HPV (*N* = 113)

	Correct	Incorrect	Don’t know
HPV is the virus that causes herpes	55 (48.7 %)	13 (11.5 %)	45 (39.8 %)
Genital warts are caused by HPV	35 (31.0 %)	32 (28.3 %)	46 (40.7 %)
HPV can cause cervical cancer	90 (79.6 %)	1 (0.9 %)	22 (19.5 %)
Regular Pap test is the best way to prevent complications caused by HPV	100 (88.5 %)	6 (5.3 %)	7 (6.2 %)
A normal Pap test means that the woman is not infected with HPV	28 (24.8 %)	54 (47.8 %)	31 (27.4 %)
Changes in Pap test may indicate infection with HPV	68 (60.2 %)	18 (15.9 %)	27 (23.9 %)
Genital warts are caused by herpes virus	22 (19.5 %)	45 (39.8 %)	46 (40.7 %)
HPV may cause cancer	92 (81.4 %)	2 (1.8 %)	19 (16.8 %)
Pap test almost always detects HPV	61 (54.0 %)	17 (15.0 %)	35 (31.0 %)
At present, there is no treatment that cures HPV	23 (20.3 %)	41 (36.3 %)	49 (43.4 %)
HPV is transmitted sexually	65 (57.5 %)	20 (17.7 %)	28 (24.8 %)
Which of the following increase risk of HPV infection?			
Initiation of sexual relations before the age of 16 years old	57 (50.4 %)	27 (23.9 %)	29 (25.7 %)
Having multiple sexual partners	91 (80.5 %)	6 (5.3 %)	16 (14.2 %)
That your partners had history of multiple sexual partners	84 (74.3 %)	10 (8.8 %)	19 (16.9 %)
Not using condoms during sexual intercourse	80 (70.8 %)	15 (13.3 %)	18 (15.9 %)

Table 3 describes factors associated to HPV and HPV vaccine awareness. Overall, 76.9 % of women had ever heard of HPV, and 57.8% were aware of the existence of HPV vaccines. Younger women (15–29 years) and those with higher educational attainment (college graduates) had increased awareness of HPV and the HPV vaccine (*p* < 0.05).

**Table 3:** Factors associated with HPV and HPV vaccine awareness

	Heard about HPV infection (*n* = 147)			Heard of HPV vaccine (*n* = 147)		
	*n*	(%)	*p* value	*n*	(%)	*p* value
Overall	113	76.9	–	84	57.8	–
Sociodemographics						
Age			0.08			0.15
≤35	66	82.5		50	62.5	
≥36	47	70.6		34	50.8	
Education			0.005			0.0002
No high school degree	10	55.6		3	16.7	
High school graduate/no college degree	40	70.2		31	54.4	
College degree or above	63	87.5		50	69.4	
IBD type			0.425			0.398
Crohn’s	63	80.8		48	61.5	
Ulcerative colitis	45	71.4		32	50.8	
Indeterminate colitis	5	83.3		4	66.7	
Current smoking			1.0			1.0
Yes	4	80.0		3	60.0	
No	109	76.8		81	57.0	
Civil status			0.546			0.628
Single	45	81.8		33	60.0	
Married/in romantic relationship	54	74.0		42	57.5	
Divorced/widow	14	73.7		9	47.4	
Employment status			0.234			0.444
Employed	46	83.6		35	63.6	
Unemployed	51	70.8		39	54.2	
Student	16	80.0		10	50.0	
Socioeconomic status			0.561			0.071
<$24,000	62	74.7		46	55.4	
$25,000–$99,000	18	90.0		16	80.0	
>$100,000	15	79.0		12	63.2	
Unknown	18	75.0		10	41.7	
Place of birth			0.474			0.235
PR	105	77.8		75	55.6	
USA	8	66.7		9	75.0	
Parity (number of live births)			0.491			0.219
0	46	79.3		37	63.8	
1–2	38	80.9		28	59.6	
≥3	29	70.7		19	46.3	
Lifetime number of sexual partners			0.882			0.657
0	15	79.0		9	47.4	
1–2	61	75.3		48	59.3	
≥3	33	80.5		24	58.5	
Age of first coitus (years)^a^			0.796			0.639
<16	20	80.0		15	60.0	
16–21	44	77.2		31	54.4	
>21	32	72.7		28	63.6	
Abnormal Pap^b^			0.834			0.994
Yes	21	77.8		16	59.3	
No	69	75.8		54	59.3	
STD history			0.069			0.559
Yes	12	100.0		8	66.7	
No	100	74.6		76	56.7	

^a^ *n* = 96; 17 patients either refused to answer or had no previous sexual relations

^b^ *n* = 90; 23 patients with no previous Pap

Among those who knew of the vaccine, 75 % learned about its existence through the media, while only 17 % through their physician/health care professional. Only 3 women of the 147 participants (2 %) had been vaccinated against HPV. This represents only 5 % compliance (three of 56 participants who were 9–26 years old) with the recommendations of the Advisory Committee for Immunization Practices [17]. Despite the low reported compliance with HPV vaccination, 50 % of women indicated that they would definitely/probably vaccinate against HPV in the future (data not shown).

Table 3 compares the bivariate association between several variables and HPV and HPV vaccine awareness. Results showed that women with higher education were more probable to be aware of HPV and HPV vaccine than women with less education (*p* = 0.005 and *p* = 0.0002, respectively). No other covariate was significantly (*p* < 0.05) associated with HPV and HPV vaccine awareness in bivariate analysis. In age-adjusted logistic regression analysis (Table 4), a significant trend was observed where more educated women were more likely to have heard of HPV (*p* for trend = 0.0017). Women who were high school graduates/some college (OR = 6.63, 95 % CI = 1.71–25.66) and those with at least an associate degree (OR = 11.69, 95 % CI = 3.05–45.89) were more likely to be aware of the HPV vaccine than women without a high school degree. No significant interaction was observed between age and educational attainment within the model (*χ*^2^ = 1.67, *p* > 0.05).

**Table 4:** Factors associated with HPV and HPV vaccine awareness

	Heard about HPV	Heard of vaccine
	OR (95 % CI)	OR (95 % CI)
Age (years)		
≤35	1.00	1.00
≥36	0.48 (0.21–1.08)*	0.62 (0.31–1.26)
Education		
Less than high school	1.00	1.00
High school/some college	1.96 (0.65–5.95)	6.63 (1.71–25.66)
Associate degree or above	5.85 (1.79–19.08)**	11.69 (3.05–44.89)**
*p* value for trend	0.0017	0.0002

*p < 0.10; *p* < 0.05

## Discussion

Patients with chronic conditions requiring immunosuppressive therapy, such as a patient with IBD, need a more structured and regular follow-up by medical providers to address different special health needs. The comprehensive management of these patients includes special attention to routine health management recommendations like vaccination. In IBD patients, HPV vaccine should be encouraged in view of the foreseeable risk of cervical cancer and the proven benefits of vaccination. It would be expected that young women with IBD were more aware of routine health maintenance medical care, like HPV vaccination and cervical cancer prevention. However, this study demonstrates low awareness about HPV and HPV vaccine in women with IBD evaluated in the UPR Center for Inflammatory Bowel Diseases. These findings are comparable with other studies in Hispanic populations. A study performed by Tortolero et al. found similar levels of awareness of HPV in Puerto Rican women over 18 years old [18]. Another study performed in Puerto Rican men who attended a sexually transmitted disease clinic showed low HPV knowledge and awareness [12]. Despite these findings, small studies have found a high prevalence of abnormal cervical cytology and high-risk HPV infection in Puerto Rican women in clinic-based samples [19]. Although no population-based studies about abnormal cervical cytology exist for Puerto Rican women, a recent population-based study among women living in the San Juan metropolitan (2010–2013) area showed that the prevalence of cervical and anal infection was 29.4 % (95 % CI 23.2–36.4 %) and 38.6 % (95 % CI 30.1–47.9 %), respectively [20]. Furthermore, the prevalence of these findings in Puerto Rican patients with IBD who are at increased risk of cervical malignancy is unknown.

The finding of poor knowledge about HPV in Hispanics or Latina women has been documented in several studies. For example, a study published by Markham et al. in Latino youth enrolled in nine alternative high schools in Houston, Texas, found that HPV knowledge was significantly lower in youths whose mothers were born outside USA versus youths of USA-born mothers [21]. In our study, of those patients who reported awareness about HPV, only a small proportion recognized that HPV could be acquired by sexual intercourse and that it may cause genital warts (57.5 and 31 %, respectively). Participants with a higher level of education reported more awareness of HPV (*p* = 0.005). Regression analysis in our study demonstrated that younger women had higher awareness of HPV and HPV vaccine. In a similar study performed by Drewry et al. among Latina immigrants aged 19 to 50 years old, 47 % of participants reported being aware of HPV [22]. This study was performed as part of a randomized clinical trial that evaluated the efficacy of a theory-based and culturally relevant intervention focusing on primary and secondary prevention of cervical cancer in women self-identified as of Latina ethnicity. They identified that women aged 40–50 years and women who had a Pap smear in the last year were more likely to report awareness of HPV. These findings provide information about which subgroup of our IBD population should be targeted for education about HPV infection risk factors and prophylaxis. Furthermore, the analysis of the specific details about HPV infection showed a significant deficit in general knowledge, emphasizing the need to develop adequate education and prevention strategies that can positively impact women with IBD who are at increased risk of HPV infection and cervical cancer. Our study showed ample knowledge about the availability of HPV vaccine in those participants that reported awareness about HPV. Although ample knowledge about the vaccine was shown in our study, only a small percentage learned this from their health-care provider and vaccination was minimal. Only three participants reported being vaccinated with HPV vaccine. This represents only 5 % compliance (three from 56 participants who were 9–26 years old) with the recommendations of the Advisory Committee for Immunization Practices [15]. Unfortunately, few studies have analyzed awareness of HPV and cervical cancer and HPV vaccination in Puerto Rico. Romaguera et al. [23] described the awareness to HPV and cervical cancer on a population-based study in 566 Puerto Rican women aged 16–64 years from a metropolitan area in Puerto Rico. They found that 64.8 % of the cohort had heard about HPV and 4.9 % of the participants with indication to receive the vaccine reported compliance with the recommended three doses. Most of the participants in this study knew about the vaccine through the media and less than half through their primary care providers. A recent qualitative study in Puerto Rican woman recruited in hospitals in San Juan and other communities found that concerns about efficacy and, to some extent, safety of HPV vaccination were associated with participants’ perception that vaccination had not been well promoted or recommended by their providers [24].

Several factors may explain the low vaccination compliance in our high-risk IBD population: insurance coverage, poor referral to vaccination programs, and limited access to vaccination programs. One of the financial barriers to widespread vaccination is that health insurance coverage of vaccination is not universal in Puerto Rico. Vaccination programs do not sponsor vaccination for patients 19–26 years old, and not all private insurances cover vaccination for patients in this same age range. Also, it is our impression that few primary providers have the infrastructure to offer vaccination in their clinics. This might be related to the increasing cost of vaccines and more strict and costly regulations required of providers, vaccination centers, and programs. Under these circumstances, appropriate referral and increasing awareness of vaccination in this population should be underscored. In our study, from 140 participants who reported reasons for not being vaccinated, almost 40 % were not aware of the existence of the HPV vaccine, 10.7 % considered not being at risk of HPV, and 5.0 % were worried of possible side effects. Teitelman et al. identified several barriers for HPV vaccination in a group of young women aged 13 to 26 years from an economically disadvantaged urban community [25]. Among the identified factors were the misconception that the vaccine might lead to long-term side effects, lack of insurance covering the HPV vaccine, and difficulties making appointments. We did not directly address barriers to vaccination in our study. It is important to characterize these barriers in order to provide adequate primary prophylaxis to this high-risk population. A recent study demonstrated that HPV vaccine elicits excellent seroconversion in women with IBD using immunosuppressive medication, with no severe adverse events [26]. A similar pilot project by our research group shows similar results (unpublished data).

This study has some limitations. This is a cross-sectional study, and no causal relationship can be established in terms of knowledge of HPV and cervical cancer risk factors and the actual risk of HPV infection and cervical cancer risk. This study included a relatively small sample of patients limiting the generalization of findings obtained in subgroup analysis. This specific limitation might be addressed in a well-designed population-based study. We had limited gynecologic information of study participants and were unable to establish the prevalence of HPV infection and cervico-vaginal abnormalities in the women that visited our clinic. In addition, we were unable to include a healthy control group to compare the findings with those of the IBD patients. However, a similar study has been performed in Puerto Rican women without IBD [18].

## Conclusion

Awareness of HPV risk factors and HPV vaccination is unsatisfactory in this underserved population. Our study illustrates this medical issue in women with IBD evaluated in the UPR Center for Inflammatory Bowel Diseases. There is a need to develop education and vaccination programs that can directly impact this high-risk population; targeted education efforts should focus on women with reduced educational attainment. Physicians and health-care workers who care for IBD patients should encourage vaccination, preferably before administration of immunosuppressive medications. Also, it is important that physicians and health-care workers who care for women with IBD encourage compliance with current endorsed cervico-vaginal cancer surveillance recommendations, based on age and risk factors. This is particularly important for gastroenterologists closely involved in the treatment of IBD. Despite the paucity of scientific data about the most appropriate surveillance in women with IBD using immunosuppressive medication, it is our opinion that this group should be considered at high risk for malignancy. Furthermore, cervical cancer surveillance may be more important in women older than 26, with no previous history of HPV immunization, who may have more cumulative exposure to immunosuppressive drugs in view of length of disease and in whom vaccination is currently not endorsed by current clinical guidelines. How cost-effective might vaccination be in women with IBD older than 26 years old is an undefined topic.
